# The Global Impact of COVID-19 on the Care of People With Endometriosis

**DOI:** 10.3389/fgwh.2021.662732

**Published:** 2021-09-29

**Authors:** Lysia Demetriou, Emma Cox, Claire E. Lunde, Christian M. Becker, Adriana L. Invitti, Beatriz Martínez-Burgo, Marina Kvaskoff, Kurtis Garbutt, Emma Evans, Elaine Fox, Krina T. Zondervan, Katy Vincent

**Affiliations:** ^1^Nuffield Department of Women's and Reproductive Health, University of Oxford, Oxford, United Kingdom; ^2^Endometriosis United Kingdom (UK), London, United Kingdom; ^3^Biobehavioral Pediatric Pain Lab, Department of Psychiatry, Boston Children's Hospital, Boston, MA, United States; ^4^Pain and Affective Neuroscience Center, Department of Anesthesiology, Critical Care, and Pain Medicine, Boston Children's Hospital, Boston, MA, United States; ^5^Departamento de Ginecologia, Universidade Federal de São Paulo, São Paulo, Brazil; ^6^Université Paris-Saclay, UVSQ, Univ. Paris-Sud, Inserm, Gustave Roussy, “Exposome and Heredity” Team, CESP, Villejuif, France; ^7^Oxford University Hosptials National Health Service (NHS) Foundation Trust, Oxford, United Kingdom; ^8^Department of Experimental Psychology, University of Oxford, Oxford, United Kingdom; ^9^Wellcome Centre for Human Genetics, University of Oxford, Oxford, United Kingdom

**Keywords:** endometriosis, COVID-19, mental health support, survey, prioritisation

## Abstract

Endometriosis is a chronic condition affecting ~10% of women globally. Little is known about the impact of the coronavirus disease 2019 (COVID-19) pandemic on their care. This brief report is aimed to explore the impact of COVID-19 on the care of people with endometriosis around the world, their priorities in relation to their clinical care during and coming out of the pandemic, and whether they believed that endometriosis makes them more vulnerable to COVID-19. An internet-based survey collected data in five languages between May 11, 2020, and June 8, 2020. Only participants with a surgical or radiological diagnosis of endometriosis aged 18 years or over were included. A total of 6,729 eligible respondents completed the survey with 80.7% [95% CI (79.7, 81.6)] reporting a negative impact on their care. This included difficulties obtaining medication (20.3%), cancelled/postponed gynaecology appointments (50.0%), and cancelled/postponed procedures (37.2%). More than half worried that their endometrioses make them more vulnerable to COVID-19 [54.2%; 95% CI (53.0, 55.4)]. The top three priorities were remarkably consistent around the world: contact with gynaecologists, knowing when procedures would be performed, and support with mental health (20.3% prioritising this aspect during the pandemic and 13.0% as restrictions begin to ease). This study shows the substantial impact the COVID-19 pandemic has had on people with endometriosis and describes how they would like care prioritised moving forwards. The findings regarding significant support needs for mental health add further weight to the growing recognition of attending to such issues as part of good patient-centred care.

## Introduction

The coronavirus disease 2019 (COVID-19) pandemic meant an abrupt change in healthcare provision around the world. Whilst the primary focus was (rightly) on the care of those infected with severe acute respiratory syndrome coronavirus 2 (SARS-CoV-2) and public health measures to prevent transmission or identify those most at risk, individuals with chronic conditions saw their treatments halted, cancelled, or changed, with little information available and extremely limited access to clinicians (1–3). Endometriosis is a chronic disease associated with pelvic pain and infertility, affecting ~10% of women and known to have high personal, societal, and financial costs (4). Due to its chronic nature, many of those affected rely on long-term medication, whilst others require one or more procedures (surgery or fertility treatment) (5, 6).

During the challenging times of the COVID-19 pandemic, the lives of people across the world have been majorly impacted. The lockdown restrictions have affected all domains of life: work routine, healthcare, education and leisure time, and for some employment or personal relationships status. Recent studies on the psychological impacts of the pandemic on the general population have shown increased psychological distress, higher than the already expected upwards trend even before the pandemic (7), and specifically a rise in depressive and anxiety symptoms (8). A recent study on the COVID-19 pandemic and endometriosis in Turkey has provided some evidence that people with endometriosis worry about the management of their condition during the pandemic (3). Additionally, several reports have been published that highlight the expected worsening of chronic pain conditions during the pandemic (9–11). Therefore, endometriosis with a chronic pain component and increased prevalence of psychological disorders could be perceived as a condition at high risk for worsening of both endometriosis-associated pain symptoms and mental health. We, therefore, considered it important to understand both how COVID-19 had affected access to care relating to endometriosis and what people with the condition would prioritise whilst the pandemic continued and as health services begin to return to a “new normal.” This study aimed to determine this on a global level.

## Methods

An online survey (JISC: https://www.jisc.ac.uk/online-surveys) was undertaken between May 11, 2020, and June 8, 2020 [Ethics Approval: The Central University Research Ethics Committee, University of Oxford (reference number R69636/RE001)]. The survey was prepared in English and translated by native speakers into French, German, Spanish, and Portuguese. The study was advertised widely on social media by researchers and clinicians in endometriosis, women and reproductive health, and relevant support groups around the world (see Acknowledgements section below). The survey (available as Supplementary Materials) assessed a variety of areas relevant to endometriosis, the COVID-19 pandemic, and the impact of the pandemic on endometriosis-associated care and symptoms. These included endometriosis symptomatology and any change after the onset of the COVID-19 pandemic, mental health, social support, access to care, and personal experience of COVID-19. Additionally, we asked what aspects of healthcare would be a priority to the participants during the COVID-19 pandemic and with the ease of lockdown restrictions.

Data were extracted and analysed in SPSS (Version 26). Responses from participants not meeting inclusion criteria (≥18 years old; self-reported diagnosis of endometriosis by surgery or imaging) were excluded, and free-text responses were translated into English by native speakers of the relevant languages and categorised by two researchers (LD and KV). The data were analysed as a single dataset (across 84 countries) and then at a continental level (Europe, North America, Latin America and Caribbean, and Oceania). Chi-squared tests were conducted to explore how the priorities of people with endometriosis might differ between the continents. Finally, the results are also presented per country for countries that each comprised more than 5% of the sample (United Kingdom, France, the USA, Brazil, Germany, and Australia). The data comprised of categorical variables thus results are presented as frequencies and percentages.

## Results

A total of 7,246 respondents completed the survey with 6,729 meeting inclusion criteria. The mean age of eligible responder was 32.5 years (range: 18–73 years). Respondents were from around the world, with the greatest proportion in Europe (Europe: *n* = 4,517; North America: *n* = 963; Latin America and Caribbean: *n* = 656; Oceania: *n* = 378; Asia: *n* = 36; Africa: *n* = 27; and unknown location: *n* = 152).

Overall, 64.6% reported no impact of the pandemic on the availability of their usual treatments for endometriosis (*n* = 4,267). However, 20.3% (*n* = 1,337) reported difficulty obtaining repeat prescriptions, 10.5% having to change their hormone and/or painkiller (4.5 and 7.0%, respectively), whilst 9.5% had to stop a medication altogether (hormones: 3.4%; painkillers: 6.6%). The impact on planned care was much greater: 50.0% of responders reported cancelled/postponed appointments with gynaecologists, and 14.7% described cancelled/postponed primary care appointments; 37.2% had procedures cancelled/postponed (surgery: 27.0% and fertility: 12.0%). Overall, 80.7% [95% CI (79.7, 81.6)] reported an impact on their current or planned treatments. These proportions were similar around the world (Tables 1, 2).

**Table 1:** Alterations to current and planned treatments.

**Alterations to treatments**	**Global** **(*****N*** **= 6,603)**		**Europe** **(*****N*** **= 4,433)**		**Oceania** **(*****N*** **= 374)**		**North America** **(*****N*** **= 961)**		**Latin America and** **Caribbean (*****N*** **= 649)**	
	**No.**	**%**	**No.**	**%**	**No.**	**%**	**No.**	**%**	**No.**	**%**
No impact	4,267	64.62	2,898	65.37	231	61.76	597	62.12	419	64.56
Difficulty with repeat prescriptions	1,337	20.25	853	19.24	105	28.07	205	21.33	127	19.57
Change hormone treatments	295	4.47	207	4.67	9	2.41	42	4.37	25	3.85
Change painkillers	459	6.95	331	7.47	29	7.75	50	5.20	34	5.24
Stop hormone treatments	222	3.36	150	3.38	7	1.87	24	2.50	32	4.93
Stop painkillers	434	6.57	295	6.65	26	6.95	55	5.72	44	6.78
**Alterations to planned treatments**	**Global** **(*****N*** **= 4,943)**		**Europe** **(*****N*** **= 3,266)**		**Oceania** **(*****N*** **= 264)**		**North America** **(*****N*** **= 676)**		**Latin America and** **Caribbean (*****N*** **= 587)**	
	**No.**	**%**	**No.**	**%**	**No.**	**%**	**No.**	**%**	**No.**	**%**
Gynaecologist appointments cancelled/postponed	2,473	50.03	1,696	51.93	88	33.33	294	43.49	321	54.68
GP appointments cancelled/postponed	726	14.69	455	13.93	27	10.23	121	17.90	96	16.35
Surgeries cancelled/postponed	1,333	26.97	840	25.72	112	42.42	222	32.84	128	21.81
Fertility treatments cancelled/postponed	591	11.96	400	12.25	25	9.47	75	11.09	76	12.95

*Global and regional results for Europe, Oceania, North America, Latin America, and Caribbean to the questions “Has the pandemic altered the availability of your treatments for endometriosis?” and “Has the pandemic altered your planned treatments relating to endometriosis?” For clarity, the table includes answers that had more than 5% frequency. Regions are defined as per the WHO recommendations (https://www.ghsindex.org/). Data presented as frequencies (No.) and percentages (%). Percentages are calculated using the number of responders in each region for each question as the denominator*.

**Table 2:** Alterations to current and planned treatments and priorities during and as restrictions ease.

	**United Kingdom**	**France**	**USA**	**Brazil**	**Germany**	**Australia**
**Date of 1st lockdown announcement**	**23rd March, 2020**	**17th March, 2020**	**19th March, 2020**	**21st March, 2020**	**23rd March, 2020**	**21st March, 2020**
**Date of 1st confirmed COVID-19 case**	**31st January, 2020**	**24th January, 2020**	**21st January, 2020**	**25th February, 2020**	**27th January, 2020**	**25th January, 2020**
	**(%)**	**(%)**	**(%)**	**(%)**	**(%)**	**(%)**
**Alterations to treatments**						
No impact	42.69	34.33	35.55	42.83	24.95	39.82
Difficulty with repeat prescriptions	25.36	12.35	18.98	22.87	16.42	28.02
Change hormone treatments	5.71	4.63	3.68	4.44	2.99	2.36
Change painkillers	7.79	9.63	4.53	5.46	5.12	8.26
Stop hormone treatments	4.60	2.00	2.12	2.56	1.49	1.77
Stop painkillers	5.76	12.72	6.09	7.34	2.35	7.67
Other	15.44	0.36	19.83	21.16	10.87	16.52
**Alterations to planned treatments**						
Gynaecologist appointments cancelled/postponed	52.56	55.79	43.85	56.31	53.31	37.24
GP appointments cancelled/postponed	16.36	8.76	19.47	16.38	12.45	10.46
Surgeries cancelled/postponed	36.20	12.29	32.38	22.98	19.46	42.26
Fertility treatments cancelled/postponed	9.84	16.53	11.07	14.12	13.23	10.88
Other	19.09	10.03	25.00	15.63	27.63	27.62
**Priorities during the pandemic**						
Mental health support	17.56	23.24	23.72	29.02	12.50	21.64
Primary care appointments	10.67	8.33	7.39	5.01	5.39	8.19
Arrange procedures	24.75	11.99	24.72	18.83	9.05	50.88
Medicine availability	8.83	11.62	9.80	4.66	39.66	20.18
Gynaecologist appointments	32.85	36.87	28.27	35.92	15.95	58.19
**Priorities as restrictions ease**						
Mental health support	8.60	12.65	14.62	18.74	19.15	14.33
Primary care appointments	4.21	3.07	3.03	3.41	2.90	5.56
Arrange procedures	49.95	37.31	51.17	26.24	26.95	52.05
Medicine availability	3.15	7.77	8.55	2.90	5.57	8.77
Gynaecologist appointments	31.69	34.69	19.45	46.34	38.53	15.79

*Results presented by country to the questions “Has the pandemic altered the availability of your treatments for endometriosis?” and “Has the pandemic altered your planned treatments relating to endometriosis?,” “During the pandemic, what one thing would be most helpful to you, relating to endometriosis?,” and “As restrictions begin to ease and healthcare starts to go back to normal, what one thing do you think should be prioritised with regards to endometriosis?” For clarity, the table includes answers that had more than 5% frequency. Data presented as percentages (%) calculated using the number of responders in each country for each question as the denominator*.

**Figure 1:**
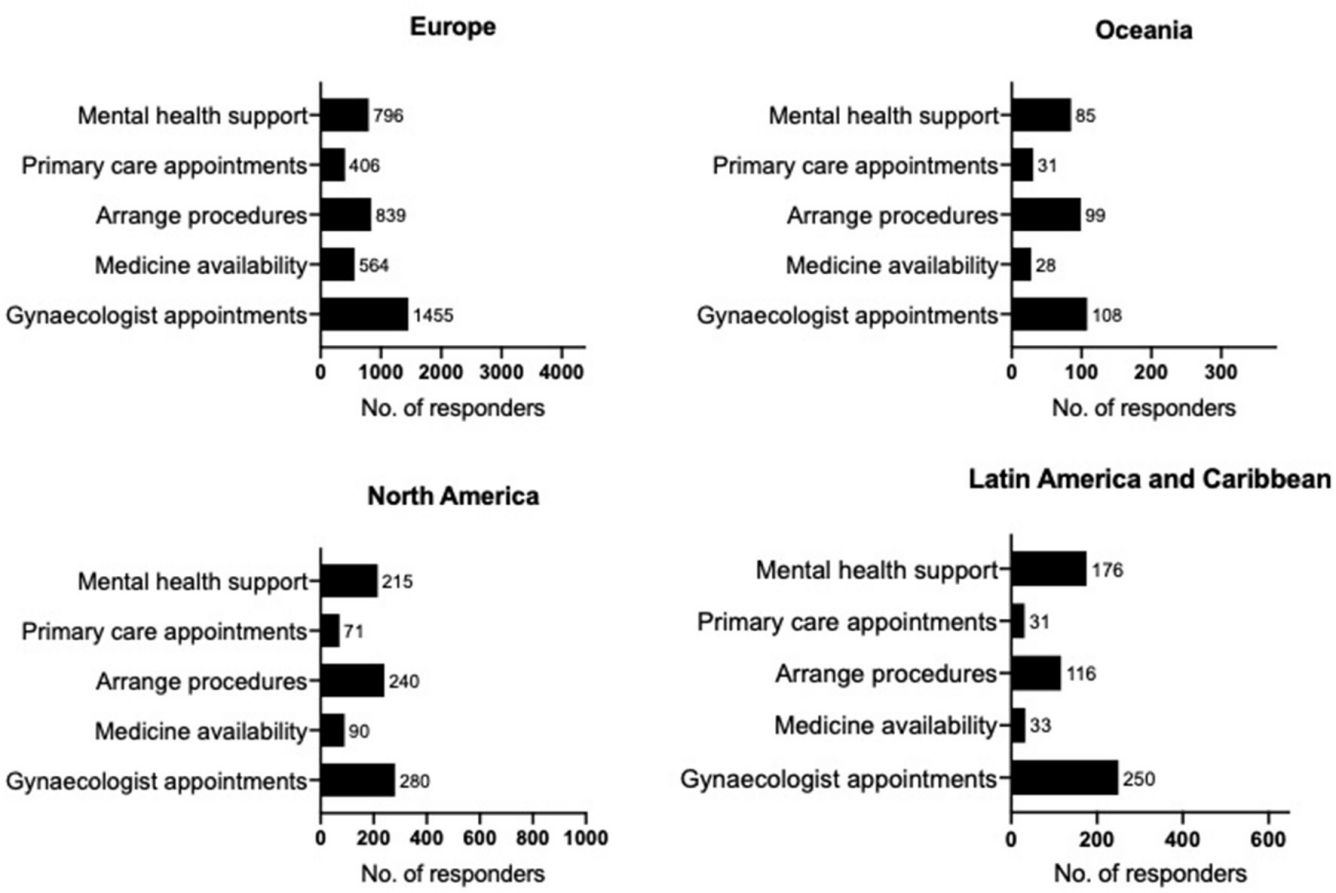
Priorities during the pandemic. Regional results for Europe (*N* = 4,398), Oceania (*N* = 370), North America (*N* = 939), Latin America, and Caribbean (*N* = 642) to the question “During the pandemic, what one thing would be most helpful to you, relating to endometriosis?**”** For clarity, the graphs include answers that had more than 2% frequency. Regions are defined as per WHO recommendations. Data presented as frequencies (No.).

**Figure 2:**
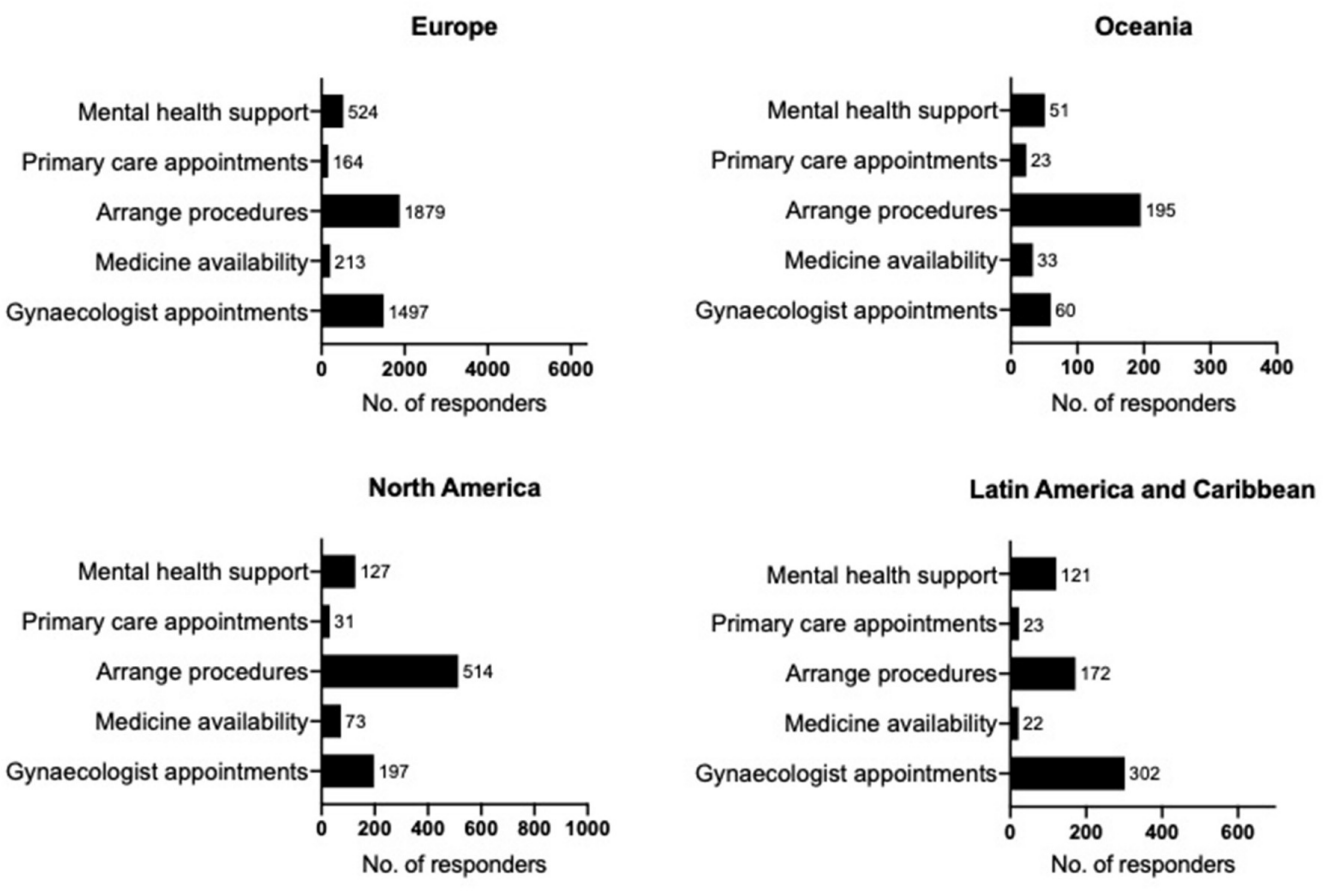
Priorities as restrictions ease. Regional results for Europe (*N* = 6,334), Oceania (*N* = 370), North America (*N* = 951), Latin America, and Caribbean (*N* = 649) to the question “*As restrictions begin to ease and healthcare starts to go back to normal, what one thing do you think should be prioritised with regards to endometriosis?”* For clarity, the graphs include answers that had more than 2% frequency. Regions are defined as per the WHO recommendations. Data presented as frequencies (No.).

Respondents considered that during the pandemic, the most helpful things would be the following: contact with their gynaecologist (32.6%), dates booked for future surgery/fertility treatments (20.5%), and mental health support (20.3%). Improving the availability of medication and contact with primary care was less popular (11.1 and 8.6%, respectively). As restrictions ease, priorities are arranging cancelled/postponed procedures (42.7%) or appointments with their gynaecologists (32.1%) and mental health support (13.0%). Considerably less chose medication availability (5.3%) or primary care appointments (3.8%). Whilst trends appear similar across regions statistical comparisons of the proportions showed significant regional variations for what the participants would find most helpful during the pandemic [$\chi_{\left(12\right)}^{2}$ = 115.0, *p* = 0.000] and once restrictions ease [$\chi_{\left(12\right)}^{2}$ = 127.8, *p* = 0.000]. Nonetheless, contact with gynaecologist, arranging procedures, and mental health support were the top three priorities across regions for both priorities during the pandemic and as restrictions ease even though the order of these three varied between regions (Figures 1, 2).

More than half of respondents worried that their endometrioses make them more vulnerable to COVID-19 [*n* = 3,635, 54.2% 95% CI (53.0, 55.4); only *n* = 22 did not answer this question].

## Discussion

Our data demonstrate the considerable impact the COVID-19 pandemic has had on the care of people around the world with endometriosis. In fact, of 6,729 eligible respondents, 5,428 (80.7%) indicated they had been affected in at least one way. It was essential that clinical practise in obstetrics and gynaecology changed during the pandemic to protect both patients and healthcare staff (12–14). However, understanding what is important to people with endometriosis will be essential as we redesign/reprioritise services.

This is the largest study of the experiences and wishes of those with endometriosis during COVID-19. A small Turkish study (*n* = 261) described 53.6% believing management of their endometriosis was affected by the pandemic with 83.9% being scared of having endometriosis-related problems during this period (3). Our findings agree with Urology and Dermatology studies, suggesting a significant impact on benign services (1, 2). Concerns have also been expressed about the impact on those with chronic pain, both in terms of difficulties accessing treatments, such as physiotherapy and psychology and the possibility of medication issues due to telephone prescribing (9).

Worldwide, respondents to our survey were remarkably consistent about the top three priorities during and immediately after the pandemic. Interestingly, the announcement of the first lockdown in the countries that we have received the most responses from happened within 7 days between March 11, 2020, and March 23, 2020. Even though the restrictions themselves differed between countries, with some going into national lockdowns whilst others introduced regional lockdowns or other less strict measures, they all saw an immediate change in healthcare services that had to adapt to deal with the new pandemic. Therefore, it is not surprising that, across countries, we see similar impacts on treatments and priorities of people with endometriosis. Whilst it was perhaps not surprising that contact with gynaecologists and knowing when procedures would be performed was important, we did not expect to see such a high proportion prioritising mental health. There has been an increasing focus on comorbid mental health conditions in people with endometriosis over recent years (15, 16); however, guidance on the management of the condition has not been updated to reflect this (5, 6). Our data have important implications on how we design services during the current infection wave and beyond. There has been a large shift toward telemedicine in all specialties (17) and ensuring the availability of gynaecologists to provide this service will be important, arguing against them being redeployed to cover emergency services as commonly occurred during the height of the first wave. It appears that talking to a primary care physician is not the priority. Given the considerable pressures placed on primary care services during the pandemic (18), this should not be recommended as a substitute for gynaecology appointments. Mental health support, on the other hand, can be delivered virtually, both standalone and in the context of pain management (11). Given the increasing prevalence of psychological distress since the onset of the COVID-19 pandemic (7, 19), it would seem prudent for healthcare providers to invest in this area for all affected not just those with endometriosis and/or chronic pain. This will be of even greater importance if concerns about increasing numbers of chronic pain patients as a consequence of this pandemic are borne out (10).

Additionally, we were concerned to see that more than 50% of those with endometriosis worried that this disease might make them more vulnerable to COVID-19. This may be because the known link to altered immunological responses has been misinterpreted as endometriosis being an autoimmune condition (4), plus additional concerns for those with thoracic endometriosis. Given that so far there is no evidence to support this belief, we consider it essential that clinicians address this issue with their patients, and education campaigns should be considered.

The size of the response over a 4-week period demonstrates the importance of this topic to those with endometriosis, and our global coverage, captured in five languages, is a strength of the study. Nonetheless, as with any survey study, there are limitations to these data. We could not access medical records to confirm the diagnosis. However, respondents who did not describe a surgical or imaging diagnosis were excluded and at the time of data collection, a face-to-face study or contact through designated hospital clinics was not a possibility. Whilst we did assess for comorbid long-term medical conditions, we did not explore how these or other aspects of the health of the participants, such as postpartum or mental health conditions, may have influenced their healthcare priorities. Additionally, it is likely that the participants may not be representative of the background population of those with endometriosis. Instead, they may represent those who interact regularly with endometriosis support groups or whose particular worries relating to their endometriosis during the pandemic had led them to visit support group sites for advice during the time our study was advertised. Nonetheless, the study was advertised globally, and we received respondents from across the globe, such as areas of the world that are usually overlooked in endometriosis studies. Given the inherent differences in healthcare systems around the world, it is perhaps surprising that we did not find more variation in the priorities of the participants or the impact of the pandemic on them. However, slight variations in the order of the top priorities between regions could be explained by differences in the healthcare systems; for example, in Latin America and the Caribbean the second highest priority is mental health support, this may reflect the large treatment gap in mental health in many of the countries in the region (20).

## Conclusions

The COVID-19 pandemic has affected the care of the majority of people with endometriosis. Moving forwards, it will be important to prioritise the components most valued by those suffering from the condition. Whilst surgery or fertility-related procedures may be challenging at times of high infection/transmission rates, contact with gynaecologists via telemedicine and the provision of remote mental health support need to be prioritised.

## Data Availability Statement

The raw data supporting the conclusions of this article will be made available by the authors, without undue reservation.

## Ethics Statement

The studies involving human participants were reviewed and approved by The Central University Research Ethics Committee, University of Oxford. The patients/participants provided their written informed consent to participate in this study.

## Author Contributions

KV conceived the study and drafted the manuscript. EC, CL, CB, AI, BM-B, MK, KG, EE, EF, KZ, and KV designed the study including translations. LD, CB, AI, BM-B, and MK analysed the data. All authors contributed to reviewed the manuscript.

## Funding

Funding for the study was provided by internal funding resources at the University of Oxford.

## Conflict of Interest

The authors declare that the research was conducted in the absence of any commercial or financial relationships that could be construed as a potential conflict of interest.

## Publisher's Note

All claims expressed in this article are solely those of the authors and do not necessarily represent those of their affiliated organizations, or those of the publisher, the editors and the reviewers. Any product that may be evaluated in this article, or claim that may be made by its manufacturer, is not guaranteed or endorsed by the publisher.
