# Supplementary material for: The Global Impact of COVID-19 on the Care of People With Endometriosis
Source: Front Glob Womens Health. 2021 Sep 29;2:662732. doi: 10.3389/fgwh.2021.662732 (PMC8594023; doi:10.3389/fgwh.2021.662732)
Supplement: Supplementary file 1 [file Data_Sheet_1.pdf]

A. Questions about your endometriosis:

1. Please tell us about your diagnosis of endometriosis. Select one of the following:

   ☐ I have had endometriosis confirmed by surgery (*e.g.* laparoscopy)
   ☐ A doctor has told me I have endometriosis, I have not had surgery
   ☐ I am undergoing investigations for endometriosis, I have not yet had a diagnosis
   ☐ I suspect I have endometriosis
   ☐ Not sure
   ☐ Not applicable
   ☐ Other (please specify)

2. How old (years) were you when you were diagnosed with endometriosis?

3. How long (years/months) had you had symptoms for before being diagnosed?

4. Which symptom was most important/bothersome to you before the pandemic? Please only tick one.

   ☐ Pelvic pain
   ☐ Heavy and/or irregular bleeding
   ☐ Fatigue
   ☐ Bowel problems
   ☐ Urinary/bladder problems
   ☐ Pain during or after sex
   ☐ Infertility/difficulty getting pregnant
   ☐ Other (please describe)

5. Were you on any treatments for your endometriosis before the pandemic? Please tick as many as apply.

   ☐ No
   ☐ Hormone tablets (*e.g*. contraceptive pills, progesterone tablets etc.)
   ☐ Hormone injections (*e.g.* contraceptive injection, menopause injections)
   ☐ Hormone coil (*e.g*. Mirena, Levosert, Jaydess, Kyleena)
   ☐ Pain killer tablets
   ☐ Pain killer injections
   ☐ Pain killer patches
   ☐ Other (please describe)

6. Were you on the waiting list before the pandemic for any of the following:

   ☐ An appointment to see a Gynaecologist
   ☐ Surgery
   ☐ Fertility treatment
   ☐ Other (please specify)

7. Have you been diagnosed with any other medical long-term medical conditions? Please tick all that apply.

**Conditions:**	**Yes**
Anxiety requiring medication or therapy	☐
Asthma	☐
Cardiovascular disease	☐
Crohn's Disease	☐
Chronic Fatigue Syndrome (CFS) / Myalgic encephalomyelitis (ME)	☐
Deafness/difficulty hearing	☐
Depression requiring medication or therapy	☐
Diabetes requiring diet control	☐
Diabetes requiring insulin or tablets	☐
Eczema	☐
Fibroids	☐
Fibromyalgia	☐
Glandular Fever	☐
Graves' disease	☐
Hashimoto's disease	☐
High blood pressure	☐
Irritable Bowel Syndrome (IBS)	☐
Migraine	☐
Mitral valve prolapse	☐
Multiple Sclerosis	☐
Painful bladder syndrome/interstitial cystitis (NOT bacterial bladder infection)	☐
Pelvic Inflammatory Disease (PID)	☐
Polycystic Ovary Syndrome	☐
Premenstrual Syndrome/Premenstrual Dysphoric Disorder	☐
Rheumatoid Arthritis	☐
Scoliosis (curvature of the spine)	☐
Spine problems (excluding scoliosis)	☐
Sjogren's syndrome	☐
SLE (Lupus)	☐
Thyroid disease	☐
Ulcerative Colitis	☐
Other (*Please specify*):	☐

B. Questions about the impact of the COVID-19 pandemic on your health:

1. Has the pandemic altered the availability of your treatments for endometriosis? Please tick all that apply.
    - ☐ No
    - ☐ It has been hard to get repeat prescriptions/medications
    - ☐ I have had to change my hormone treatment
    - ☐ I have had to change my painkillers
    - ☐ I have had to stop all hormone treatments
    - ☐ I have had to stop some/all my painkillers
    - ☐ Other (please describe)

2. Has the pandemic altered your planned treatments relating to endometriosis? Please tick all that apply.
    - ☐ I was awaiting an appointment with my GP/Family Dr and I haven't been given one yet
    - ☐ I had an appointment with my GP/Family Dr and it has been cancelled
    - ☐ I was awaiting an appointment with a Gynaecologist and I haven't been given one yet
    - ☐ I had an appointment with a Gynaecologist and it has been cancelled
    - ☐ I was awaiting a date for surgery and I haven't been given one yet
    - ☐ I had a date for my surgery and it has been cancelled
    - ☐ I was awaiting a date for fertility treatment and I haven't been given one yet
    - ☐ I had a date for starting my fertility treatment and it has been cancelled
    - ☐ I had started fertility treatment and this has been halted
    - ☐ Other – please describe

3. Has the pandemic altered the availability of treatments for any of your other medical conditions?
    - ☐ Not applicable
    - ☐ No
    - ☐ Yes – please describe

4. Compared to 6 months ago, how would you describe what has happened to your endometriosis-associated pain since the pandemic was announced?
    - ☐ Very marked worsening
    - ☐ Marked worsening
    - ☐ Minimal worsening
    - ☐ No change
    - ☐ Minimal improvement
    - ☐ Marked improvement
    - ☐ Very marked improvement
    - ☐ I didn't experience any pain due to endometriosis and this hasn't changed

5. Compared to 6 months ago, how would you describe what has happened to your tiredness/fatigue since the pandemic was announced?
    - ☐ Very marked worsening
    - ☐ Marked worsening
    - ☐ Minimal worsening
    - ☐ No change
    - ☐ Minimal improvement
    - ☐ Marked improvement
    - ☐ Very marked improvement
    - ☐ I didn’t suffer with tiredness/fatigue and this hasn’t changed

6. Compared to 6 months ago, how would you describe what has happened to your bleeding pattern since the pandemic was announced? Please tick the sentence that best describes your experience:
    - ☐ I don’t have periods/vaginal bleeding and this hasn’t changed
    - ☐ I did have regular periods but my period hasn’t come during the pandemic (and I am not pregnant)
    - ☐ I did have regular periods and my period was unusually late during the pandemic
    - ☐ I did have regular periods and my period has continued to come on time
    - ☐ I did have regular periods but I have had a lot of unexpected bleeding during the pandemic
    - ☐ I did have regular periods but I have bled every day/almost every day during the pandemic
    - ☐ I did have irregular bleeding and my bleeding has become more frequent during the pandemic
    - ☐ I did have irregular bleeding but I have bled every day/almost every day during the pandemic
    - ☐ I did have irregular bleeding and my bleeding has become less frequent during the pandemic
    - ☐ I did have irregular bleeding but I haven’t bled at all during the pandemic
    - ☐ I didn’t bleed at all but I have had irregular bleeding during the pandemic
    - ☐ I didn’t bleed at all but I have bled every day/almost every day during the pandemic
    - ☐ Other – please describe

7. Compared to 6 months ago: please tick all that apply
    - ☐ I haven’t changed my hormone treatments
    - ☐ I have changed the hormone treatment I am using (for endometriosis or contraception)
    - ☐ I am no longer pregnant
    - ☐ I am now pregnant
    - ☐ I am no longer breast feeding
    - ☐ I am now breast feeding

8. Please rate the pelvic pains you have been experiencing during the pandemic on a 0-10 scale where 0=no pain and 10=worst pain imaginable:

   1. Pain with your periods/bleeding (0-10, I don't have periods/bleeding drop-down list)
   2. Pain with passing urine/weeing (0-10 drop-down list)
   3. Pain with opening your bowels/pooing (0-10 drop-down list)
   4. Pain with sex (0-10, I don't have sex drop-down list)
   5. Pain that is present most/every day but not related to any of the above triggers (0-10 drop-down list)

9. Please answer the following questions in the context of what is possible during the pandemic by ticking one box for each statement.

   For example: how has your pain impacted your ability to socialize *virtually?*

**In the past 7 days…**	**Not at all**	**A little bit**	**Somewhat**	**Quite a bit**	**Very much**
How much did pain interfere with your enjoyment of life?					
How much did pain interfere with your ability to concentrate?					
How much did pain interfere with your day to day activities?					
How much did your pain interfere with your enjoyment of recreational activities?					
How much did pain interfere with doing your tasks away from home (*e.g.* getting groceries, running errands)?					
How often did pain keep you from socializing with others?					

10. Everyone experiences painful situations at some point in their lives. Such experiences may include headaches, tooth pain, joint, or muscle pain. People are often exposed to situations that may cause pain such as illness, injury, dental procedures, or surgery.

    We are interested in the types of thoughts and feelings that you have when you are in pain. Listed below are thirteen statements describing different thoughts and feelings that may be associated with pain. Using the scale, please indicate the degree to which you have these thoughts and feelings when you are experiencing pain related to your endometriosis.

	Not at all	To a slight degree	To a moderate degree	To a great degree	All the time
I worry all the time about whether the pain will end	☐	☐	☐	☐	☐
I feel I can't go on	☐	☐	☐	☐	☐
It's terrible and I think it's never going to get any better	☐	☐	☐	☐	☐
It's awful and I feel that it overwhelms me	☐	☐	☐	☐	☐
I feel I can't stand it anymore	☐	☐	☐	☐	☐
I become afraid that the pain will get worse	☐	☐	☐	☐	☐
I keep thinking of other painful events	☐	☐	☐	☐	☐
I anxiously want the pain to go away	☐	☐	☐	☐	☐
I can't seem to keep it out of my mind	☐	☐	☐	☐	☐
I keep thinking about how much it hurts	☐	☐	☐	☐	☐
I keep thinking about how badly I want the pain to stop	☐	☐	☐	☐	☐
There's nothing I can do to reduce the intensity of the pain	☐	☐	☐	☐	☐
I wonder whether something serious may happen	☐	☐	☐	☐	☐

11. How has the pandemic altered your mental health?
    - ☐ Very marked worsening
    - ☐ Marked worsening
    - ☐ Minimal worsening
    - ☐ No change
    - ☐ Minimal improvement
    - ☐ Marked improvement
    - ☐ Very marked improvement
    - ☐ Unsure

12. Please answer these questions by marking one box per row:

In the past 7 days...	Never	Rarely	Sometimes	Often	Always
I felt worthless					
I felt that I had nothing to look forward to					
I felt helpless					
I felt sad					
I felt like a failure					
I felt depressed					
I felt unhappy					
I felt hopeless					
I felt fearful					

In the past 7 days...	Never	Rarely	Sometimes	Often	Always
I felt fearful					
I found it hard to focus on anything other than my anxiety					
My worries overwhelmed me					
I felt uneasy					
I felt nervous					
I felt like I needed help for my anxiety					
I felt anxious					
I felt tense					

C. Questions about your experience of the COVID-19 pandemic:

1. Have you had symptoms of COVID-19? yes/no/not sure

2. Have you had a positive test for COVID-19? yes/no

3. Have you been admitted to hospital because of COVID-19? yes/no

4. Has anyone you live with had symptoms of COVID-19? yes/no

5. Has anyone you live with had a positive test for COVID-19? yes/no

6. Has someone close to you (family or friend) died because of COVID-19? yes/no

7. Are you considered "vulnerable"/at high risk from COVID-19 according to a Government definition? Yes/no

8. Do you live with someone who is considered "vulnerable"/at high risk from COVID-19 according to a Government definition? Yes/no

9. Have you worried that your endometriosis makes you more vulnerable to COVID-19? yes/no

10. Has the COVID-19 pandemic led to any major life changes for you other than those experienced by everyone (*e.g.* restrictions on your life, working from home where possible, home-schooling etc.)? Please tick all that apply.
    - ☐ No, nothing more than for most people
    - ☐ Yes, I have lost my job
    - ☐ Yes, I have had to work much longer hours
    - ☐ Yes, I have had a significant decrease in my earnings
    - ☐ Yes, I can't run my business
    - ☐ Yes, I have had to move out of my home
    - ☐ Yes, my relationship with my partner has fallen apart
    - ☐ Yes, I have had to postpone/cancel my wedding
    - ☐ Other – please describe

11. Please answer these questions by marking one box per row, thinking about how things have been since the pandemic was announced.

	Never	Rarely	Sometimes	Usually	Always
I have someone who will listen to me when I need to talk.					
I have someone to confide in or talk to about myself or my problems.					
I have someone who makes me feel appreciated.					
I have someone to talk with when I have a bad day.					
I have someone who understands my problems.					
I have someone I trust to talk with about my feelings.					
I have someone with whom to share my most private worries and fears					
I have someone I trust to talk with about my problems					

12. Please answer these questions by marking one box per row, thinking about how you have felt since the pandemic was announced:

Since the pandemic was announced, how often have you…	Never	Almost never	Someti mes	Fairly often	Very often
been upset because of something that happened unexpectedly?					
felt that you were unable to control the important things in your life?					
felt nervous and "stressed"?					
felt confident about your ability to handle your personal problems?					
felt that things were going your way?					
found that you could not cope with all the things that you had to do?					
been able to control irritations in your life?					
felt that you were on top of things?					
been angered because of things that happened that were outside of your control?					
felt difficulties were piling up so high that you could not overcome them?					

D. Questions about what you think would be most helpful for you:

1. During the pandemic, what one thing would be most helpful to you, relating to endometriosis?
    - Being able to talk to my GP/family doctor
    - Being able to talk to my gynaecologist
    - Being able to have all the medicines I had before
    - Knowing when my surgery/fertility treatment will happen
    - Having some help with my mental health
    - Other (please describe)

2. As restrictions begin to ease and healthcare starts to go back to normal, what **one** thing do you think should be prioritised with regards to endometriosis?
    - Appointments with GPs/family doctors
    - Appointments with gynaecologists
    - Arranging all operations that were cancelled/postponed
    - Arranging all fertility treatments that were cancelled/postponed/might now be needed
    - Making medicines easily available again
    - Mental health support (counseling, psychology and/or medicines)
    - Other – please describe

E. Questions about you:

1. How old (in years) are you?

2. What term best describes your work status before the pandemic?

- ☐ Working in a paid job, as a full time employee or worker
- ☐ Working in a paid job, as a part time employee or worker
- ☐ Self-employed
- ☐ Not in paid work force:

3. How would you describe your ethnic origin?

☐American Indian or Alaskan native
☐Asian: ☐East Asian
☐Southeast Asian
☐South Asian
☐Black: ☐Black African
☐African American
☐Black Caribbean
☐Arab/Persian
☐Native Hawaiian or other Pacific Islanders
☐White: ☐North/West European
☐East European
☐South European
☐North American
☐Other White *(please specify)*
☐Multiple Races
☐Other *(please specify)*

4. Do you consider yourself to be Spanish/Hispanic/Latina? Yes/no

5. What is the highest level of education you have attained?

☐Primary/grade school
☐Lower secondary/middle school
☐Upper secondary/high school
☐Post-secondary not university / some college or vocational school
☐University
☐Postgraduate

6. Where do you live?
    a. Country:
    b. City:
    c. Postcode/zip code:

7. Who are you living with?

    a. Alone
    b. Flatmates/Roommates /friends
    c. Parents/family
    d. Partner, no children
    e. Partner and children
    f. Children no other adults
    g. Other – please describe

8. Are you pregnant at the moment? yes/no/unsure
    If yes, how many weeks:

Thank you for taking the time to answer these questions. The results of this study will be analysed and reported on as soon as possible to help us understand how we can reduce the impact of COVID-19 on people with endometriosis.
